# The performance of homopolymer detection using dichromatic and tetrachromatic fluorogenic next-generation sequencing platforms

**DOI:** 10.1186/s12864-024-10474-0

**Published:** 2024-05-31

**Authors:** HuiJuan Chen, Bing Wang, LiLi Cai, YiRan Zhang, YingShuang Shu, Wen Liu, Xue Leng, JinCheng Zhai, BeiFang Niu, QiMing Zhou, ShuNan Cao

**Affiliations:** 1 Beijing ChosenMed Clinical Laboratory Co. Ltd, Beijing, 100176 China; 2 Computer Network Information Center, Chinese Academy of Sciences, Beijing, 100190 China; 3 WillingMed Technology Beijing Co., Ltd, Beijing, 100176 China; 4 ChosenMed Technology (Zhejiang) Co. Ltd, Zhejiang, 311103 China; 5 Polar Research Institute of China, Shanghai, 201209 China

**Keywords:** Next-generation sequencing, Sequencing platforms, Homopolymer detection

## Abstract

**Objectives:**

Homopolymer (HP) sequencing is error-prone in next-generation sequencing (NGS) assays, and may induce false insertion/deletions and substitutions. This study aimed to evaluate the performance of dichromatic and tetrachromatic fluorogenic NGS platforms when sequencing homopolymeric regions.

**Results:**

A HP-containing plasmid was constructed and diluted to serial frequencies (3%, 10%, 30%, 60%) to determine the performance of an MGISEQ-2000, MGISEQ-200, and NextSeq 2000 in HP sequencing. An evident negative correlation was observed between the detected frequencies of four nucleotide HPs and the HP length. Significantly decreased rates (*P* < 0.01) were found in all 8-mer HPs in all three NGS systems at all four expected frequencies, except in the NextSeq 2000 at 3%. With the application of a unique molecular identifier (UMI) pipeline, there were no differences between the detected frequencies of any HPs and the expected frequencies, except for poly-G 8-mers using the MGI 200 platform. UMIs improved the performance of all three NGS platforms in HP sequencing.

**Conclusions:**

We first constructed an HP-containing plasmid based on an *EGFR* gene backbone to evaluate the performance of NGS platforms when sequencing homopolymeric regions. A highly comparable performance was observed between the MGISEQ-2000 and NextSeq 2000, and introducing UMIs is a promising approach to improve the performance of NGS platforms in sequencing homopolymeric regions.

**Supplementary Information:**

The online version contains supplementary material available at 10.1186/s12864-024-10474-0.

## Background

With the rapid development of sequencing platforms and bioinformatic algorithms, next-generation sequencing (NGS) technologies have been widely used in various areas of clinical practice, which drives the fast progression of “personalized” medicine, especially in precision oncology [1–4]. One of the greatest technical challenges of NGS is accurately detecting high-complexity regions, such as repetitive DNA sequences. DNA homopolymer (HP) tracts, also known as mononucleotide microsatellites, are sequences consisting of a series of consecutive identical bases, such as poly(dA).poly(dT) and poly(dG).poly(dC), which are the simplest of the simple sequence repeats in the genome [5]. HPs are present in all genomes, and there are probably 1.43 million HPs in the human genome, most of which are short sequence lengths (4 mers < *N* < 6 mers). In the human genome, poly(dA).poly(dT) HP tracts are overrepresented in comparison to poly(dG).poly(dC) tracts [5, 6]. Inaccuracies in detecting the HP regions often lead to the inaccurate detection of genomic variations. HP errors are prone to length change mutations, such as insertion and deletion mutations (indels) [7–9].

Multiple factors may impact the accurate identification of homopolymeric regions, including the underlying principle, sequencing chemistry, and fluorescent labeling approach of sequencing platforms; the length and nucleotide type of the homopolymeric regions; and the relative position of the homopolymeric region in the genome. Relatively high error rates of HP sequencing have been reported with certain sequencing platforms, such as in pyrosequencing and ion semiconductor sequencing platforms [10, 11]. It has been reported that the average correct genotyping results were 95.8%, 87.4%, and 72.1% for 4-mer, 5-mer, and 6-mer HPs using pyrosequencing platforms, and the accuracy decreased as the HP length gradually increased [10]. In a clinical setting, the analytical validity of the designed approach based on pyrosequencing was acceptable for 4- to 6-mer HPs, but not for 7-mers and beyond [10]. Several bioinformatic algorithms for correcting the HP sequencing errors or separating artifact variations caused by HPs from true genetic variations have been developed [11–13], and they can significantly improve the detection accuracy of pyrosequencing and ion semiconductor sequencing platforms when sequencing homopolymeric regions. Homopolymer sequencing errors seem to be overcome by the unique sequencing technologies used in the Illumina NGS platforms. However, high insertion/deletion errors caused by HPs were observed with the GS Junior (Roche), GS FLX+ (Roche), and PGM (Thermo Fisher) systems, and high substitution errors induced by HPs were found with Illumina NGS systems (MiSeq and HiSeq) [14]. It is still very important to determine the capability of the NGS system to accurately and stably detect HP regions before using the corresponding NGS system in routine clinical practice. To date, no empirical research has been reported to compare and evaluate the capability of HP region detection between different NGS platforms in clinical settings using the same dataset.

Here, a comprehensive empirical study was conducted to compare and evaluate the performance of different NGS platforms in HP sequencing. A synthesis plasmid consisting of 2- to 8-mer HPs of all four nucleotides was used to assess performance of the NextSeq 2000 (dichromatic fluorogenic platform), MGISEQ-200 (dichromatic fluorogenic platform), and MGISEQ-2000 (tetrachromatic fluorogenic platform) for HP sequencing.

## Results

### Description of the constructed template plasmid

**Fig. 1:**
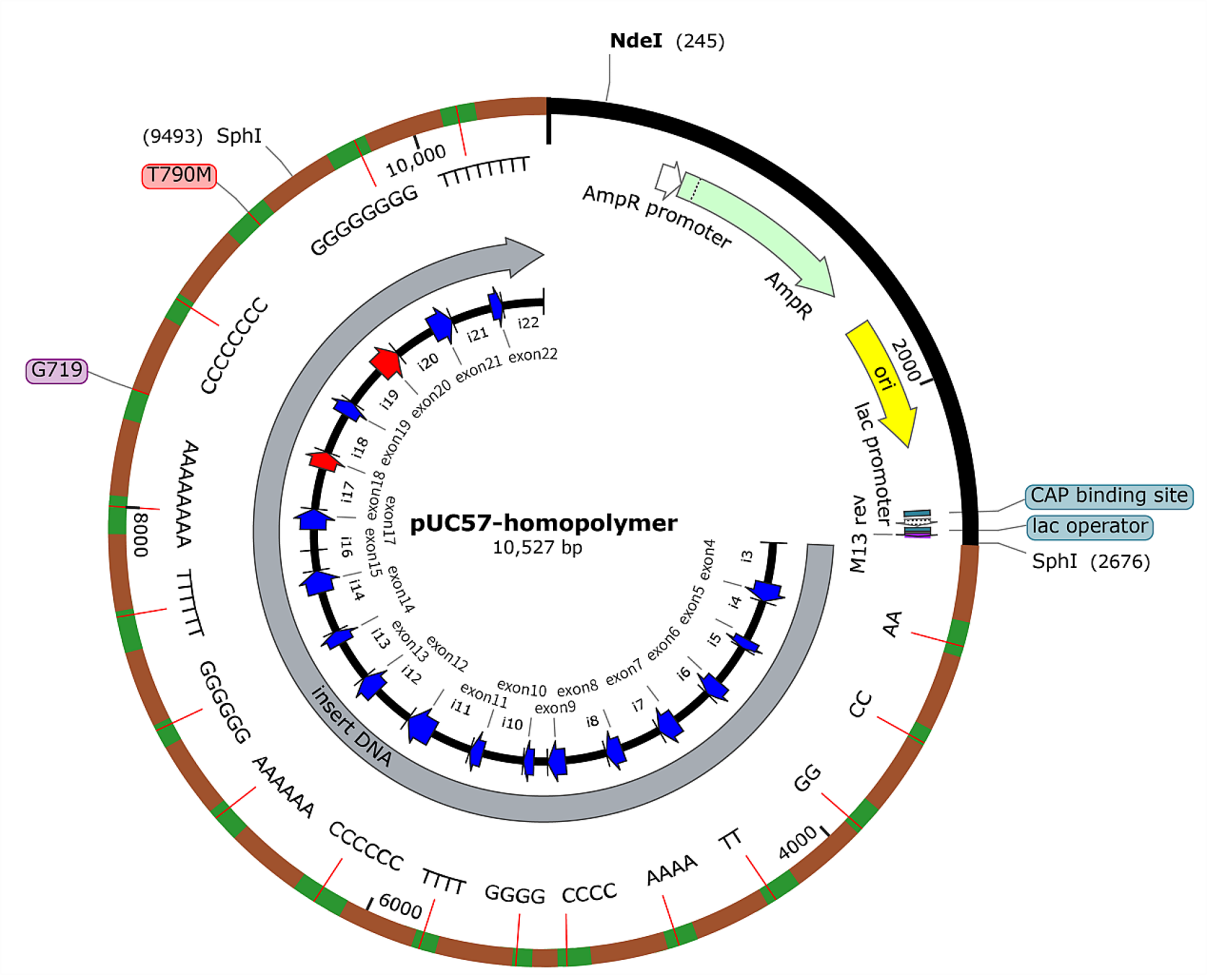
Visualization of the structure of the pUC57-homopolymer plasmid

The structure of the constructed HP-containing plasmid, a pUC57-homopolymer plasmid, is elaborated in Fig. 1. The total length of the target sequence was 7,817 bp, including the entire *EGFR* 4–22 exon regions and ± 150 bp intron regions of each exon. The exact sequence of the designed target sequence was showed in the supplementary material. The 2-mer HPs (AA, CC, GG, TT) were inserted in exons 4, 5, 6, and 7, respectively; 4-mer HPs (AAAA, CCCC, GGGG, TTTT) were inserted in exons 8, 9, 10, and 11, respectively; 6-mer HPs (AAAAAA, CCCCCC, GGGGGG, TTTTTT) were inserted in exons 12, 13, 14, and 15, respectively; and 8-mer HPs (AAAAAAAA, CCCCCCCC, GGGGGGGG, TTTTTTTT) were inserted in exons 17, 19, 21, and 22, respectively. There were no HPs inserted into exon 16 because of its short length (39 bp). The wild-type G719 in exon 18 was used as an internal reference for the quantification of the constructed plasmid. The constructed hotspot mutation T790M in exon 20 was used as the internal reference during sequencing.

### Performance of MGI and Illumina platforms in homopolymer sequencing

**Fig. 2:**
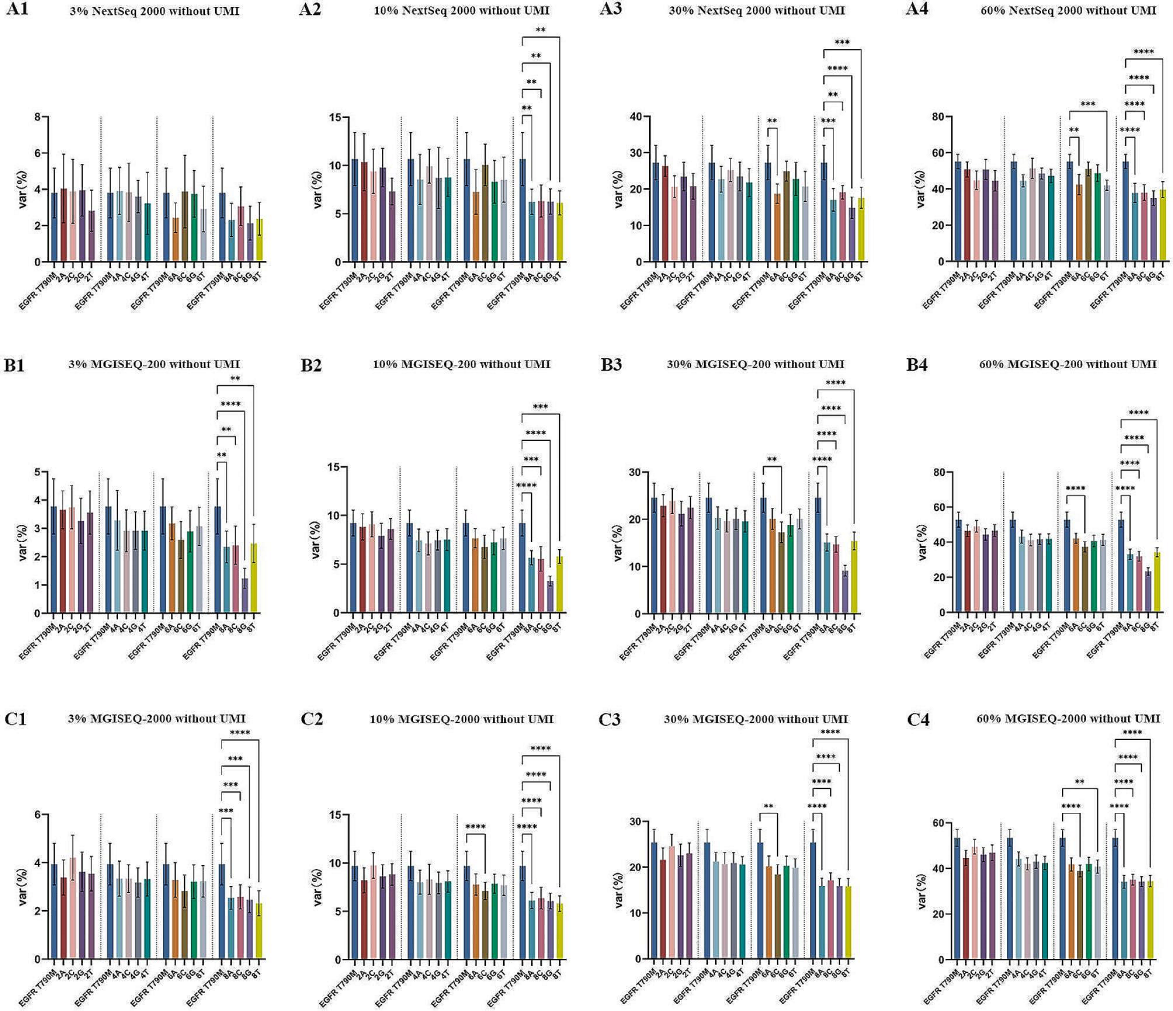
The detected frequencies of homopolymers according to nucleotides and length. The detected variant allele frequencies of T790M were used as the real expected frequencies of HPs. Var (%) represents the detected frequencies of T790M and HPs.**: *P* < 0.01; ***: *P* < 0.001; ****: *P* < 0.0001

The identical libraries of 48 HP-containing plasmids with four theoretical frequencies (3%, 10%, 30%, 60%) were sequenced using the three NGS platforms. The sequencing data were analyzed using a bioinformatic pipeline without unique molecular identifier (UMI). There were discrepancies but no statistics significant differences in the detected variant allele frequencies (VAFs) of T790M by all three NGS platforms at all four theoretical frequencies (Table 1, Table S1). Thus, the corresponding detected VAFs of T790M in each plasmid were used as the actual expected frequencies of HPs to minimum variations from sequencing. The average detected frequencies of HPs, according to nucleotides and length of HPs, are shown in Fig. 2. The average detected frequency of each HP was compared to the corresponding detected VAF of T790M.

**Fig. 3:**
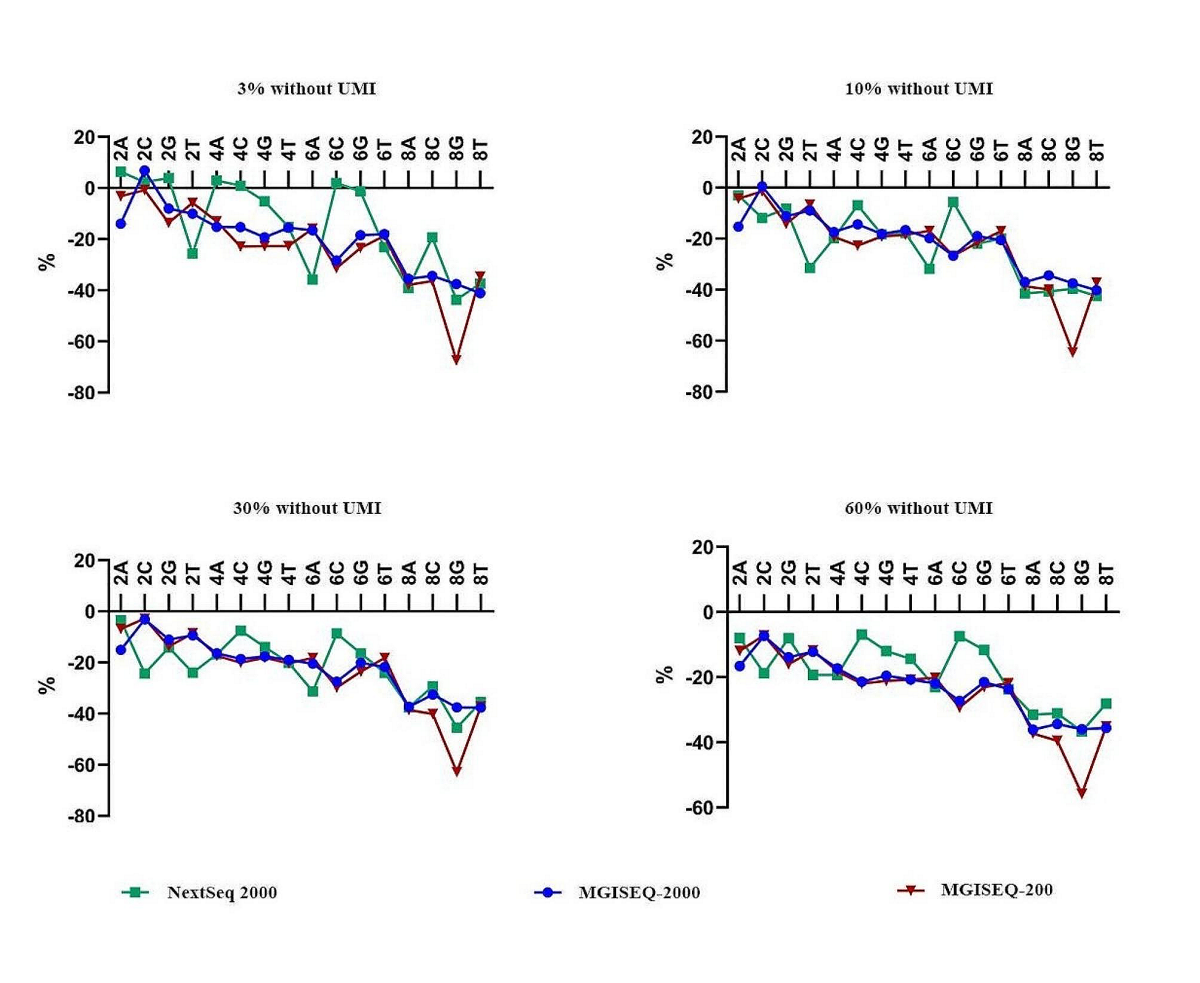
Performance comparison of three NGS platforms for homopolymer sequencing. %= ( detected frequency of HPs- detected frequency of T790M)/detected frequency of T790M ×100%

The detected frequencies of the four-nucleotide HPs decreased as the length of the HPs increased in all three NGS platforms at all four theoretical frequencies. While, incorrect base calling (mainly incorrect length) ratios in the homopolymeric regions were increased as the homopolymer length increased (Figure S1). Significantly decreased rates (*P* < 0.01) were found for all the 8-mer HPs in all three NGS systems at all four theoretical frequencies, except for the NextSeq 2000 at 3% (Fig. 2), and the MGISEQ-200 platform that demonstrated dramatically decreased rates of poly-G 8-mers (Fig. 3, Figure S2-O, S3-O, S4-O, S5-O). With the NextSeq 2000, significantly decreased rates (*P* < 0.01) were found for poly-A 6-mers at 30% (Fig. 2-A3), and for poly-A 6-mers and poly-T 6-mers at 60% (Fig. 2-A4) theoretical frequencies. Meanwhile, significantly decreased rates (*P* < 0.01) were observed in poly-C 6-mers at 30% and 60% theoretical frequencies in MGISEQ-200 platform (Fig. 2-B3, 2-B4), and in poly-C 6-mers at 10%, 30% and 60% theoretical frequencies (Fig. 2-C2, 2-C3, 2-C4), poly-T 6-mers at 60% theoretical frequency (Fig. 2-C4) in MGISEQ-2000 platform. The detected frequencies of all homopolymers were similar to the theoretical frequencies in the MGISEQ-2000, and MGISEQ-200 platforms, except for the poly-G 8-mers of MGISEQ-200; There were some discordance between MGISEQ-200 and NextSeq 2000 platforms for many of the HP sequence (Fig. 3, Figure S2-5). Our results demonstrated a highly comparable performance between the MGISEQ-2000 and NextSeq 2000 for HP sequencing.

**Table 1:** The average detected VAFs of T790M in the HP-containing plasmid by three NGS platforms at four theoretical frequencies

Platforms	3%		10%		30%		60%	
	Without UMI	With UMI	Without UMI	With UMI	Without UMI	With UMI	Without UMI	With UMI
NextSeq 2000	3.80%	4.20%	10.65%	10.69%	27.28%	27.74%	55.14%	56.77%
MGISEQ-200	3.78%	3.99%	9.22%	10.05%	24.56%	26.38%	52.86%	55.78%
MGISEQ-2000	3.94%	4.13%	9.70%	10.43%	25.39%	27.14%	53.43%	56.24%

### Unique molecular identifiers improve the performance of all NGS platforms when sequencing homopolymers

**Fig. 4:**
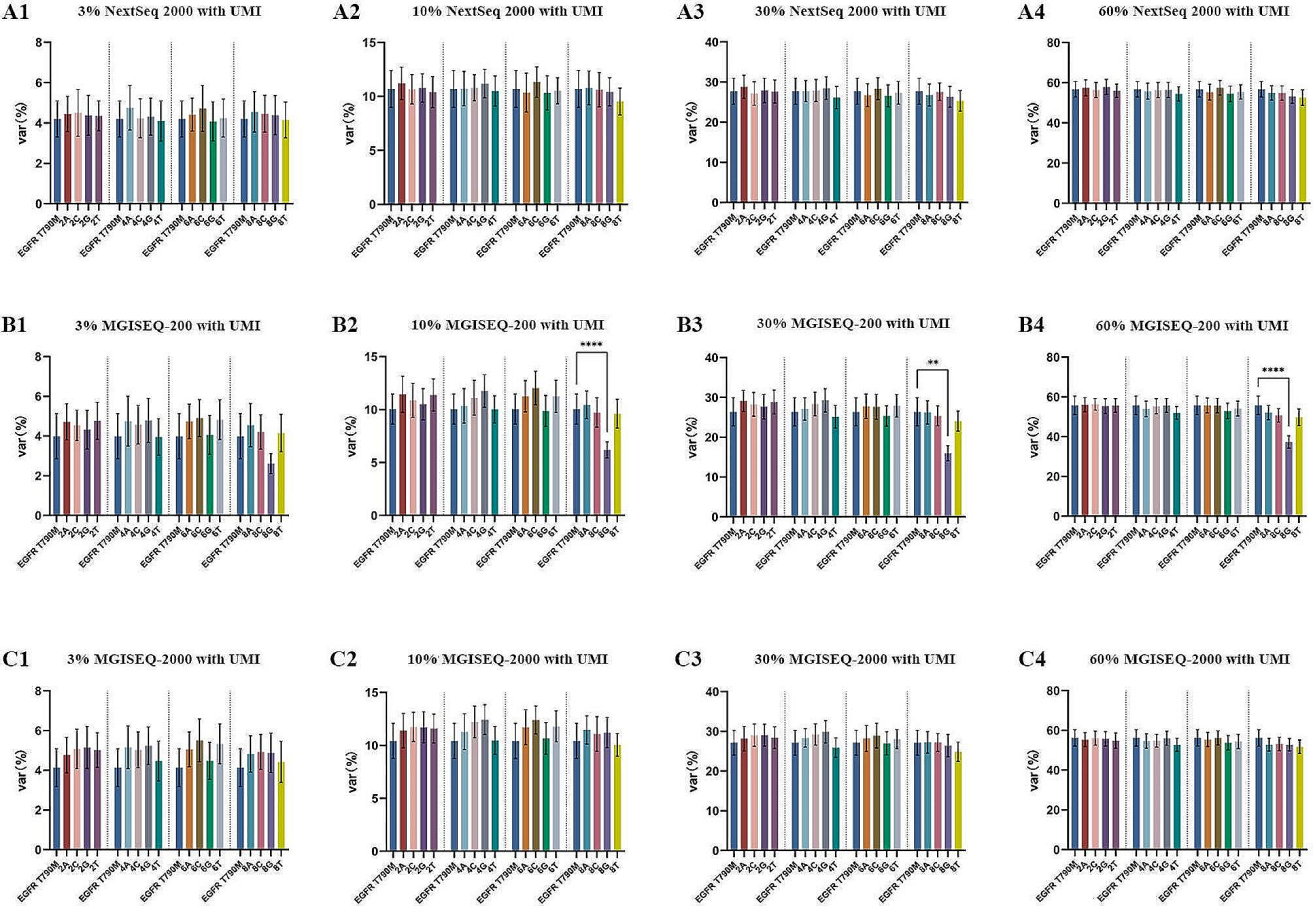
Unique molecular identifiers improve the performance of NGS platforms in homopolymer sequencing. Var (%) represents the detected frequencies of T790M and HPs. **: *P* < 0.01; ****: *P* < 0.0001

The same sequencing data were analyzed using a UMI tools embedded bioinformatic pipeline. With the application of the UMI pipeline, there were no differences between the detected frequencies of any HPs and the real expected frequencies (the detected VAF of the corresponding T790M in the same sample), except for the poly-G 8-mers using the MGISEQ-200 platform (Fig. 4). UMIs improved the performance of all three NGS platforms in HP sequencing.

**Fig. 5:**
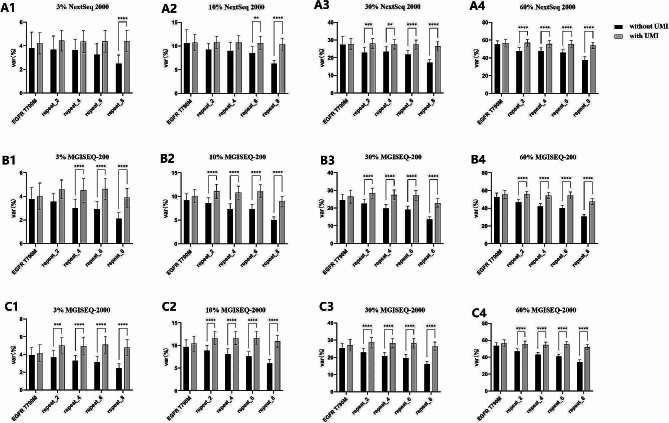
Comparison of the detected frequencies of HPs with and without using a UMI pipeline. Var (%) represents the detected frequencies of T790M and HPs. **: *P* < 0.01; ***: *P* < 0.001; ****: *P* < 0.0001

The detected frequencies of each HP and the corresponding T790M in the same sample using the UMI pipeline were compared to those without using the UMI pipeline (Fig. 5). For T790M, there were no differences with or without the UMI pipeline at all four expected frequencies using all three NGS platforms. Significant differences (*P* < 0.01) were observed for 8-mer HPs at a 3% theoretical frequency (Fig. 5-A1), 6-mer and 8-mer HPs at a 10% theoretical frequency (Fig. 5-A2), and 2-mer, 4-mer, 6-mer, and 8-mer HPs at 30% and 60% theoretical frequencies (Fig. 5-A3, A4) in the NextSeq 2000 platform. Significant differences were found for all 2-mer, 4-mer, 6-mer, and 8-mer HPs at all four theoretical frequencies using the MGISEQ-2000 and MGISEQ-200 platforms, except for 2-mers at 3% with the MGISEQ-200 (Fig. 5-B1-4; Fig. 5-C1-4).

## Discussion

Various short-read sequencing systems, such as Illumina, Ion Torrent, and MGI NGS platforms, have been introduced and are widely used in basic research and clinical fields. Several previous studies demonstrated that NGS platforms had platform-specific sequencing errors [14–16]. Some sequence-specific errors have been discovered and corrected, such as the accumulation of T fluorophores [17] in Illumina sequencing systems, software to correct this error was then developed [18]. HP sequencing is error-prone and liable to increase insertion/deletion errors and Ns. High error rates in HP regions were found with Ion Torrent platforms and Roche NGS systems [14, 19], and the Ion Torrent could not identify the correct number of bases in HPs greater than 8 bp [20]. Illumina NGS platforms were relatively more accurate when detecting HPs, due to their sequencing chemistry and technology. However, dramatically higher error rates of reads containing long HPs (> 8 bp), especially reads with long G/C HPs, were observed with the Illumina MiSeq and HiSeq platforms [14]. The performance of NGS platforms is improving with the rapid progress of sequencing techniques developed by various companies and institutes in recent years.

Illumina NGS platforms are the most popular NGS systems globally for their high throughput and accuracy. In recent years, a series of NGS platforms, such as the MGISEQ-200 and MGISEQ-2000, have been launched by MGI and are widely used in mainland China. Several studies demonstrated the high consistency between MGI and Illumina sequencing platforms in various applications, such as target capture sequencing [21], whole-genome sequencing [22], whole-exome sequencing [23], transcriptome sequencing [24], and DNA methylation [25]. In this study, we compared the performance of the MGISEQ-2000, MGISEQ-200, and NextSeq 2000 for sequencing HP regions. Differences among the three sequencing platforms include sequencing approaches and distinct fluorescent labels. Both MGISEQ-2000 and MGISEQ-200 employ sequencing based on DNA nanoball (DNB) and probe-anchor synthesis (cPAS) technology [21]. NextSeq 2000 is based on sequencing by synthesis with a fluorescent labeled reversible terminator technology. Homopolymers sequencing error is reduced by this technique due to incorporation of single base at a time, as for addition of another base terminator needs to be removed first. However, prior to sequencing, NextSeq 2000 uses bridge PCR technology to clonal amplified (cluster generation) of DNA libraries, which have a cumulative effect on sequencing errors to lead to sequence-specific errors patterns. While, MGISEQ-2000 and MGISQ-200 achieve single-molecule template amplification by DNB circularization amplification. Four-channel sequencing system is used in MGISEQ-2000, wherein each nucleotide is labeled with a distinct fluorescent dye and detected by an individual image. While, the MGISEQ-200 and NextSeq 2000 are two-channel sequencing system, which utilize two-color fluorescent labeling to determine all four nucleotides: one label for C, another label for T, both labels for A, and no label for G. The differences between MGISEQ-200 and NextSeq 2000 sequencing techniques include the fluorescent dyes. The MGISEQ-200 utilizes a red fluorescent dye label for C and green for T, while blue is used for C and green for T in the NextSeq 2000.

In order to assess the performance of these two-channel and four-channel NGS sequencing systems for sequencing homopolymeric regions, we first constructed an HP-containing plasmid based on the *EGFR* backbone (Fig. 1). This plasmid contained the most common HPs (2-mer to 8-mer) observed in clinical practice. The plasmid was diluted by 12 different *EGFR* wildtype genes to four expected frequencies, as described in the template preparation, to maximally simulate real-world samples. An obvious negative correlation was observed between reliability and HP length in all NGS platforms. We found that all three NGS systems were less reliable in the case of 8-mer HPs. MGI platforms demonstrated decreased reliability with 6-mer G/C HPs. Decreased reliability was observed with 6-mer A/T HPs in the NextSeq 2000 system, which was different than the MiSeq and HiSeq platforms [14]. This may be caused by the different fluorescent labeling approaches. Similar to the MGISEQ-2000, MiSeq and HiSeq platforms are four-channel sequencing systems.

Some strategies have been developed to correct or mitigate the sequencing errors induced by HPs in the ion semiconductor sequencing platforms [11, 12] and Roche NGS systems [19, 26, 27]. However, there are fewer strategies for other NGS platforms. UMI is a promising approach to handle errors generated during sequencing. UMIs, short random oligonucleotides, can be incorporated into template DNA molecules during an initial PCR [28] or prior to PCR [29], allowing PCR amplification and sequencing errors to be identified and corrected by a bioinformatics pipeline. UMIs, which can improve accuracy and sensitivity, are widely used in liquid biopsies for sensitive detection of circulating tumor DNA by deep sequencing [30–32] and in other fields, such as forensic genotyping [33] and immune repertoire sequencing [34]. Our study showed that UMIs improved the performance of the MGISEQ-2000, MGISEQ-200, and NextSeq 2000 for sequencing HP regions. Introducing UMIs in routine NGS testing is a good approach for improving the accuracy of NGS platforms in HP regions.

## Conclusions

In this study, we first constructed an HP-containing plasmid based on an *EGFR* gene backbone, which contained 2-mer, 4-mer, 6-mer, and 8-mer of all four nucleotide HPs, to evaluate the performance of MGISEQ-2000, MGISEQ-200, and NextSeq 2000 platforms when sequencing homopolymeric regions. Our results showed that a highly comparable performance was observed between the MGISEQ-2000 and NextSeq 2000, and UMIs significantly improved the performance of all three evaluated platforms when sequencing homopolymeric regions.

## Materials and methods

### Homopolymer-containing plasmid design and construction

Based on the backbone of the *EGFR* gene, we designed the target sequence consisting of 2-mer, 4-mer, 6-mer, and 8-mer HPs of all four nucleotides (A, G, C, T). We directly inserted the sequences of all the 2-mer, 4-mer, 6-mer and 8-mer HPs of all four nucleotides into the designated regions of *EGFR* reference sequence to generate the target sequence. Noteworthy, there was one mutation site, T790M, in the target sequence (Fig. 1). Then, the target sequence was synthesized by Sangon Biotech (Shanghai, China). The synthetic target sequence with 2- to 8-mer HPs of all four nucleotides was assembled into a pUC57 vector to form a new plasmid, which was used as a template for the following experiments.

### Template preparation

The constructed, HP-containing plasmid was linearized by NdeI restriction endonuclease (NEB, MA, USA). In order to maximize the simulation of real-world samples, the linearized plasmid DNA and the healthy volunteers DNA were detected and quantified by digital PCR. The statuses of G719 and T790 in the *EGFR* gene of DNA from the healthy volunteers were confirmed using digital PCR to ensure the *EGFR* wild-type. The copy numbers of the *EGFR* gene in the linearized plasmid and healthy volunteer DNA were quantified using the digital PCR based on the *EGFR* wild-type G719 DNA fragment. The linearized plasmid DNA was serially diluted with *EGFR* wild-type DNA from 12 healthy volunteers to generate 3%, 10%, 30%, and 60% variant allele frequency (VAF) HP-containing templates based on the copy numbers. A total of 48 HP-containing templates with expected HP VAFs were generated for subsequent NGS sequencing. This study was carried out following The Code of Ethics of the World Medical Association (Declaration of Helsinki) for experiments involving humans and it was approved by the Ethics Committee of Jinshan hospital (No. JIEC 2022-S27). Written informed consent following approved guidelines was obtained from each participant.

### Library construction and sequencing

A total of 50 ng of HP-containing template DNA was used for pre-capture library preparation with a Rapid MaxDNA Lib Prep kit (ABclonal Technology, Wuhan, China) through the following sequential steps: ultrasonic fragmentation, end repair and A-tailing, ligation and low-cycle amplification. In detail, a total of 50 ng of plasmid DNA was sheared using the Covaris M220 focused-ultrasonicator (Covaris, MA, the USA) to generate fragments with an average size of 150–300 base pairs; The end repair and A-tailing master mix was prepared as follow: 7 µl rapid max end prep buffer, 3 µl end prep enzymes, 50 ng sheared DNA, water to a final volume 60 µl. The end repair and A-tailing program (30℃, 30 min; 65℃, 30 min; 4℃, holding) was performed on Verity Thermocycler (Applied Biosystems, CA, USA); For adapter ligation, each 110 µl single reaction contained 60 µl end repair and A-tailing products, 30 µl rapid max ligation buffer, 10 µl ligation enzymes, 5 µl adapters (with or without UMI), 5 µl ddH_2_O. The thermal cycler program was 20℃, 30 min; The adapters in the Rapid MaxDNA Lib Prep kit were regular adapters without UMIs. The UMI adapters were purchased from iGeneTech (iGeneTech, BJ, China). After ligation, excess adapters and adapter dimers were removed using one Ampure clean-up with 110 µl AmPure XP beads (1.0× volume); For libraries PCR amplification, each 50 µl single PCR reaction included 20 µl purified ligation products, 5 µl 10×PCR primers, 25 µl 2×PCR master mix. PCR reactions were performed in 0.2 µl thin-wall microtubes on a Verity Thermalcycler with the following conditions: 98℃ for 45 s, 8 cycles of 98℃ 15 s, 60℃ 30 s, 72℃ 30 s, a final extension at 72℃ for 1 min. After PCR, excess primers and primer dimers were removed using one Ampure clean-up with 50 µl AmPure XP beads (1.0× volume).

The pre-capture library was captured using a custom-designed ChosenOne® NGS 27 gene lung panel (ChosenMed, Beijing, China), which covered the entire coding sequence (CDS) and part of the introns of the *EGFR* gene. Each library was divided into three parts, and parallel sequenced on the MGISEQ-200 with control software suite ECR4.2 (BGI, Shenzhen, China), MGISEQ-2000 with control software suite ECR6.0 (BGI, Shenzhen, China), and NextSeq 2000 with control software suite version 1.5.0.42699 (Illumina, San Diego, CA, USA) with 100-bp paired-end cycles.

### Bioinformatic analyses

All of the raw sequence data from the three NGS platforms were analyzed using the bioinformatic analysis pipelines, with and without UMI processing (Figure S6, Figure S7). The raw sequence data cleaned using fastp (v0.22) [35], which filtered out the adapter contamination reads, low-quality reads, low complexity sequences. (BWA, v0.7.17)-MEM [36] and SAMtools [37] were applied for alignment with the reference genome hg19, and generating SAM/BAM files. Picard (v1.119) was incorporated to mark the duplicate reads. For the bioinformatic pipeline with UMI, a set of Fgbio tools, such as fgbio correctUmis, fgbio GroupReadsByUMI, were introduced for the UMI processing (Figure S3). Genome Analysis TK (GATK, v4.2.6.1) [38] was for quality score-based re-calibration and indel realignment. Single nucleotide variations and indels were identified by Vardict (v1.8.2) [39] and VarScan [40]. The specific HP sequences were identified by an in-house developed bioinformatic script, HomopolymerFinder. We are providing this scrip freely available on GitHub at https://github.com/lilicai/HomopolymerFinder. Picard and fgbio are freely available on Github at http://broadinstitute.github.io/picard/index.html and https://github.com/fulcrumgenomics/fgbio, respectively. All the parameters setting of each step of two bioinformatic pipelines were introduced in detail in Table S4.

### Statistics

All of the data were analyzed using GraphPad Prism, version 9.1.0 (GraphPad Software, San Diego, CA). Variables between multiple groups were investigated by one-way ANOVA (normal distribution data) or non-parametric tests (non-normal distribution data). *P* < 0.01 was considered to be statistically significant.

### Electronic supplementary material

Below is the link to the electronic supplementary material.

Supplementary Material 1

Supplementary Material 2

Supplementary Material 3

Supplementary Material 4

Supplementary Material 5

Supplementary Material 6

Supplementary Material 7

Supplementary Material 8

Supplementary Material 9

Supplementary Material 10

## Data Availability

The datasets generated and/or analysed during the current study are available in the Genome Sequence Archive (GSA) repository, accession number: PRJCA020397 (https://ngdc.cncb.ac.cn/gsa-human/browse/HRA005779).
