# Supplementary material for: The performance of homopolymer detection using dichromatic and tetrachromatic fluorogenic next-generation sequencing platforms
Source: BMC Genomics. 2024 May 31;25:542. doi: 10.1186/s12864-024-10474-0 (PMC11140927; doi:10.1186/s12864-024-10474-0)
Supplement: Supplementary file 1 — Supplementary Material 1 [file 12864_2024_10474_MOESM1_ESM.docx]

>EGFR HP DNA target sequence

acatgcatatcatttatgctgtgaccactgactaaaccattctcttccttcctccccatatttctaaatttctaatcattgctcaaagcccaattcagagaaaaccctagctcctccatggcaccatcattaacaattttatctggccgccccccgggaagttcactgggctaattgcgggactcttgttcgcaccatggcatctctttagcagaacataaatgcgaagagcacatgcatccttcatgggaatttaaaggagctggaaagagtgctcaccgcagttccattctcccgcagAAATCCTGCATGGCGCCGTGCGGTTCAGCAACAACCCTGCCCTGTGCAACGTGGAGAGCATCCAGTGG**AA**CGGGACATAGTCAGCAGTGACTTTCTCAGCAACATGTCGATGGACTTCCAGAACCACCTGGGCAGCTgtaagtgtcgcatacacactatctctgcctccagctcctatgggggacagctctacagcactggggcaggggagagaagccatgtttagtaagtcacattaatcagaaacaaaaagtagtaagcaaaatatctgaccactagaaaagcatgattctacaaaccagccagccaaacaatcagagaataagttgaaaagattgtcttcatttattgaatgtgcttaactcaggcccgggaaagggcgtcatcagtttctcatcatttcactgagatatgcatctattacttttacatttcagGCCAAAAGTGTGATCCAAGCTGTCCCAATGGG**CC**AGCTGCTGGGGTGCAGGAGAGGAGAACTGCCAGAAACgtaagtcagtgaacagcctcagacccatgtgtgaccgcccctctcttccttcacttgcttaggtgattggatttgttttccctctgaagactccaaagagttactttattacagggtcagatgtgaaccagtaggtgaaggacagtcttggcatggtttgacttagtttgaatgtggtttcgttggaagcaaatgtgtcttcactttttcatgaaaaagtctgcaagtgctctgcgacatccctgggaaatgatcctaccctcactcttcagctcacagggaacctttgctctttttcagTGACCAAAATCATCTGTGCCCAGCAGTGCTCCGGGCGCTGCCGTGGCAAGTCCCCCAGTGACTGCTGCCA**GG**CAACCAGTGTGCTGCAGGCTGCACAGGCCCCCGGGAGAGCGACTGCCTGgtaagatgcccctccagcagcctccctggagcaggctggggctgcacccgccccacccacaccaggacagaagacttcctgtgggggagctgtcaattagcatttgtcataacagacaggatattgccctctgcctggtgacaaagtatcagctgggttttcccacactagtggaacactaggctgcaaagacagtaacttgggctttctgacgggagtcaacaccgtgctgcgcttcctccgtgtgtggcgctgagtgtacttacctcacttgcccagcgtgtcctctctcctccatagGTCTGCCGCAAATTCCGAGACGAAGCCACGTGCAAGGACACCTGCCCCCCACTCATGCTCTACAACCCCACCACGTACCA**TT**GATGGATGTGAACCCCGAGGGCAAATACAGCTTTGGTGCCACCTGCGTGAAGAAGTGTCCCCgtgagtcctcctctgtgggccctctaactggtcaggcatccttgtcccgctctgtctcctgctgagccctggagtatcccatcttggagagtctttgggtggatgtgtttgccttgcttggaggaggcgaccctgtgcccgtccaggcactgcaacctttcattctttgtccttaaagtaaataaagccaaaggaggatggagcctttccatcacccctcaagaggacctggaccgcctgtgtgaggcccgagcacctggtgccaccgtcatcaccttcctttcatgctctcttccccagGTAATTATGTGGTGACAGATCACGGCTCGTGCGTCCGAGCCTGTGGGG**AAAA**CCGACAGCTATGAGATGGAGGAAGACGGCGTCCGCAAGTGTAAGAAGTGCGAAGGGCCTTGCCGCAAAGgtaggaagcccgccggtgtgcggacgaggcttgttctcggctgctgaggctgggctctcatgccacctccaaaggaacacatcttcctcttctcattaaaaaacaactatacatatcgtttctttaaaacagaagataaagctgtaaagcccttcagtgtttgttgagtgaatgaaggatgatgtggcagtggcggttccggtgaccggaattccttcctgcttccctctgcctgtggatccctagctattcttaatccaacaaatgtgaacggaatacacgtctctcttatctctgcagTGTGTAACGGAATAGGTATTGGTGAATTTAAAGACTCACTCTCCATAAATGCTACGAATATT**CCCC**AAACACTTCAAAAACTGCACCTCCATCAGTGGCGATCTCCACATCCTGCCGGTGGCATTTAGGGGgtgagtcacaggttcagttgcttgtataaagaaaaacaaaatctgcctttttaactggtagagattggtgatcaataatcaccctgttgtttgtttcagTGACTCCTTCACACATACTCCTCCTCTGGATCC**GGGG**ACAGGAACTGGATATTCTGAAAACCGTAAAGGAAATCACAGgtttgagctgaattatcacatgaatataaatgggaaatcagtgttttagagagagaacttttcgacatatttcctgttcccttggaataaaaacatttcttctgaaattttaccgttaatggctgatgttttgatatttttcaaaagtgccagagaagatgataatgaaaaagaaagcaaatccaattttcccacttactgttcatataatacagagtccctgagagtctagagtaatgtctcatacaaaaaagaaactcctacgtggtgtgtgtctgaagtctttcatctgccttacagGGTTTTTGCTGATTCAGGCTTGGCCTGAAAACAGGAC**TTTT**GGACCTCCATGCCTTTGAGAACCTAGAAATCATACGCGGCAGGACCAAGCAACAgtaagttgaccacagccaaagcctggtagattacatttgcctttttagttggaaattaggcttaacaggagagttgctaagatagggcacagagctcctgcatctctcgccggcattcccaaatgctatctcacatgagcaggcacagggaaaggtgcagtgtgtgcctcccacagcatgacctaccatcattggaaagcagtttgtagtcaatcaaaggtggtctggagaaacaaagttttcagggatacattgtttttataatttttcaccacatgatttttcttctctccaatgtagTGGTCAGTTTTCTCTTGCAGTCGTCAGCCTGAACATAACATCCTTGGGATTACGCTCCCTCAAGGAGATAAGTGATGGAGATGTGAT**CCCCCC**AATTTCAGGAAACAAAAATTTGTGCTATGCAAATACAATAAACTGGAAAAAACTGTTTGGGACCTCCGGTCAGAAAACCAAAATTATAAGCAACAGAGGTGAAAACAGCTGCAgtaagtcaccgctttctgtttagtttatggagttggttctaatgggtcctttatttgtatttagaatattgaagggctattcccatttaaattacttttttcagttccttaagaagcaaattaaaatcttaagattcctaactgtgaaatgtctgtgtcctcctccttcaggggtagccagcatgtctgtgtcacccaaggtcatggagcacagggcccctcccgggaaggtgccgtctcctccggcccctcgggtccctgctctgtcactgactgctgtgacccactctgtctccgcagAGGCCACAGGCCAGGTCTGCCATGCCTTGTGCTCCCCCGAGGGCTGCTGGGGCCCGGAGCCCAGGGACTGCGTCTC**AAAAAA**TTGCCGGAATGTCAGCCGAGGCAGGGAATGCGTGGACAAGTGCAACCTTCTGGAGGGgtaggaggttatttctttaatccccttgcgttgatcaaaaataaggctccaggttgttgttatagctttacaggcattctgtttgattttctcttccttttattctttgcccttggcttttggaggttttgggttttctgtggggagacgacaaaatcagctgattatattactatatagtcctggagtcccaactccttgaccattacctcaagttatttggaattttgaagaggtgatttgtgttcctgcaataatgtctcaggggtgggctgacgggtttcctcttcctcctctcagTGAGCCAAGGGAGTTTGTGGAGAACTCTGAGTGCATACAGTGCCA**GGGGGG**CCCAGAGTGCCTGCCTCAGGCCATGAACATCACCTGCACAGGACGGgtaagagccccttgctgctatccacgtccatttcatgggaagggccttcacagaagccgaacagtgatgatggcccagggcatcctgtgtgggcaggacggccatcagagccacttcccagaggagacggcaggcgctgacagcgctgtcacagaataactggttttctcctttaagaatttttctatcatttggctttccccactcacacacactaaatattttaagtaaaaagttacttccattttgaaagagaaaagaaagagacatgcatgaacatttttctccaccttggtgcagGGACCAGACAACTGTATCCAGTGTGCCCACTACATTGACGGCCCCCACTGCGTCAAGACCTGCCCGGCAGGAGTCATGGGAGAAAACAACACCCTGGTCTGGAAGTACGCA**TTTTTT**GACGCCGGCCATGTGTGCCACCTGTGCCATCCAAACTGCACCTACGGgtgagtggaaagtgaaggagaacagaacatttcctctcttgcaaattcagagatcaaaaatgtctcccaagttttccggcaacaaattgccgaggtttgtatttgagtcagttacttaaggtgttttggtccccacagccatgccagtagtttctccttttagaagctacatagtgtctcactttccaagatcattctacaagatgtcagtgcactgaaacatgcaggggcgtgttgagtgccaaggccatggaatctgtcagcaacctcacccttccttgttcctccacctcattccagGCCTAAGATCCCGTCCATCGCCACTGGGATGGTGGGGGCCCTCCTCTTGCTGCTGGTGGTGGCCCTGGGGATCGGCCT**AAAAAAAA**CTTCATGCGAAGGCGCCACATCGTTCGGAAGCGCACGCTGCGGAGGCTGCTGCAGGAGAGGGAGgtgagtgccagtcctgggtgggctcaggagccctcgcaccccgacaggaacaagggccagccccgagaacgggccattagcagttgtgtatgttagatacataattgtattatgatgcagaaagaatctctgaatgtgcagttatacccatgtccttccaaatgagctggcaagtgccgtgtcctggcacccaagcccatgccgtggctgctggtccccctgctgggccatgtctggcactgctttccagcatggtgagggctgaggtgacccttgtctctgtgttcttgtcccccccagCTTGTGGAGCCTCTTACACCCAGTGGAGAAGCTCCCAACCAAGCTCTCTTGAGGATCTTGAAGGAAACTGAATTCAAAAAGATCAAAGTGCTGG**G(G719)**CTCCGGTGCGTTCGGCACGGTGTATAAGgtaaggtccctggcacaggcctctgggctgggccgcagggcctctcatggtctggtggggagcccagagtccttgcaagctgtatatttccatcatctactttactctttgtttcactgagtgtttgggaaactccagtgtttttcccaaccagcaatatcagccttaggtgcggctccacagccccagtgtccctcaccttcggggtgcatcgctggtaacatccacccagatcactgggcagcatgtggcaccatctcacaattgccagttaacgtcttccttctctctctgtcatagGGACTCTGGATCCCAGAAGGTGAGAAAGTTAAAATTCCCGTCGCTATCAAGGAA**CCCCCCCC**TTAAGAGAAGCAACATCTCCGAAAGCCAACAAGGAAATCCTCGATgtgagtttctgctttgctgtgtgggggtccatggctctgaacctcaggcccaccttttctcatgtctggcagctgctctgctctagaccctgctcatctccacatcctaaatgttcactttctatgtctttccctttctagctctagtggtaggtcttttgcaggcacagcttttcctccatgagtacgtattttgaaactcaagatcgcattcatgcgtcttcacctggaaggggtccatgtgcccctccttctggccaccatgcgaagccacactgacgtgcctctccctccctccagGAAGCCTACGTGATGGCCAGCGTGGACAACCCCCACGTGTGCCGCCTGCTGGGCATCTGCCTCACCTCCACCGTGCAGCTCATCA**T(T790M)**GCAGCTCATGCCCTTCGGCTGCCTCCTGGACTATGTCCGGGAACACAAAGACAATATTGGCTCCCAGTACCTGCTCAACTGGTGTGTGCAGATCGCAAAGgtaatcagggaagggagatacggggaggggagataaggagccaggatcctcacatgcggtctgcgctcctgggatagcaagagtttgccatggggatatgtgtgtgcgtgcatgcagcacacacacattcctttattttggattcaatcattctttggatcagtagtcactaacgttcgccagccataagtcctcgacgtggagaggctcagagcctggcatgaacatgaccctgaattcggatgcagagcttcttcccatgatgatctgtccctcacagcagggtcttctctgtttcagGGCATGAACTACTTGGAGGACCGTCGCTTGGTGCACCGCGACCTGGCAGCCAGGAACGTACTGGTGAAAACACCGCAGCATGTCAAGATC**GGGGGGGG**ACAGATTTTGGGCTGGCCAAACTGCTGGGTGCGGAAGAGAAAGAATACCATGCAGAAGGAGGCAAAgtaaggaggtggctttaggtcagccagcattttcctgacaccagggaccaggctgccttcccactagctgtattgtttaacacatgcaggggaggatgctctccagacattctgggtgagctcgcagcagctgctgctggcagctgggtccgggaggaggcgccgggcctgggggacgggtcctggggtgatctggctcgtctgtgtgtgtcactcgtaattaggtccagagtgagttaactttttccaacagagggaaactaatagttgtctcactgcctcatctctcaccatcccaagGTGCCTATCAAGTGGATGGCATTGGAATCAATTTTACACA**TTTTTTTT**GAATCTATACCCACCAGAGTGATGTCTGGAGCTACGgtgagtcataatcctgatgctaatgagtttgtactgaggccaagctggcttttattgttagttaatttacattatatcctctgacatgcaagtattttctttcgagataatgactaatgataatgtaatcattgctgtctatctattgtactgagaaaacacggcagaggaaatcgagtccagctgccgtccaaaagtcactggagattgcaatgagctcgtctggcagggtggggggtatgggagggaaagagcttaggaaacggctctccctgcaaagtccaaccaaactttaacgtt
