# Supplementary figures and images for: The performance of homopolymer detection using dichromatic and tetrachromatic fluorogenic next-generation sequencing platforms
Source: BMC Genomics. 2024 May 31;25:542. doi: 10.1186/s12864-024-10474-0 (PMC11140927; doi:10.1186/s12864-024-10474-0)

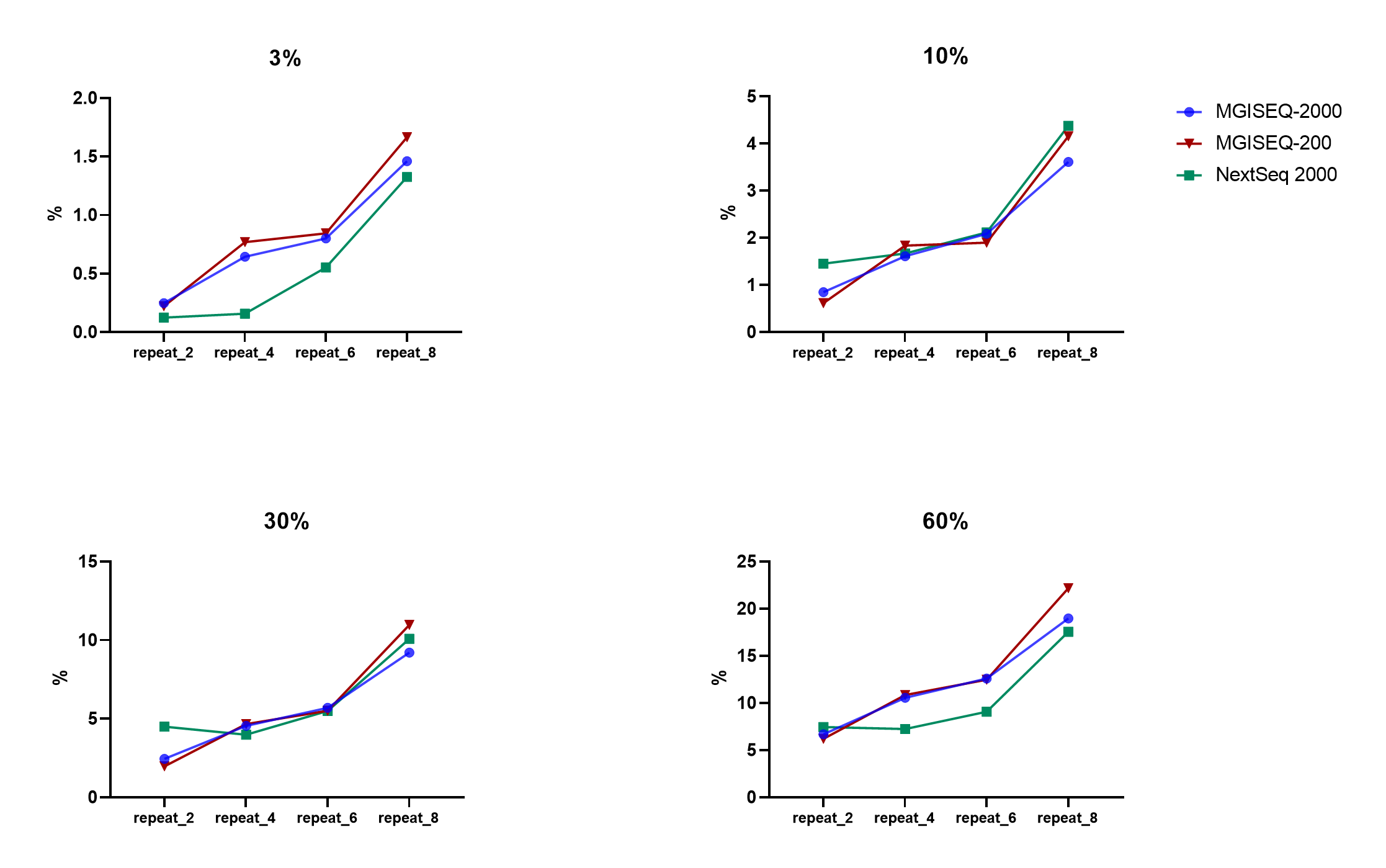

Supplement: Supplementary file 2 — Supplementary Material 2 [file 12864_2024_10474_MOESM2_ESM.tif]

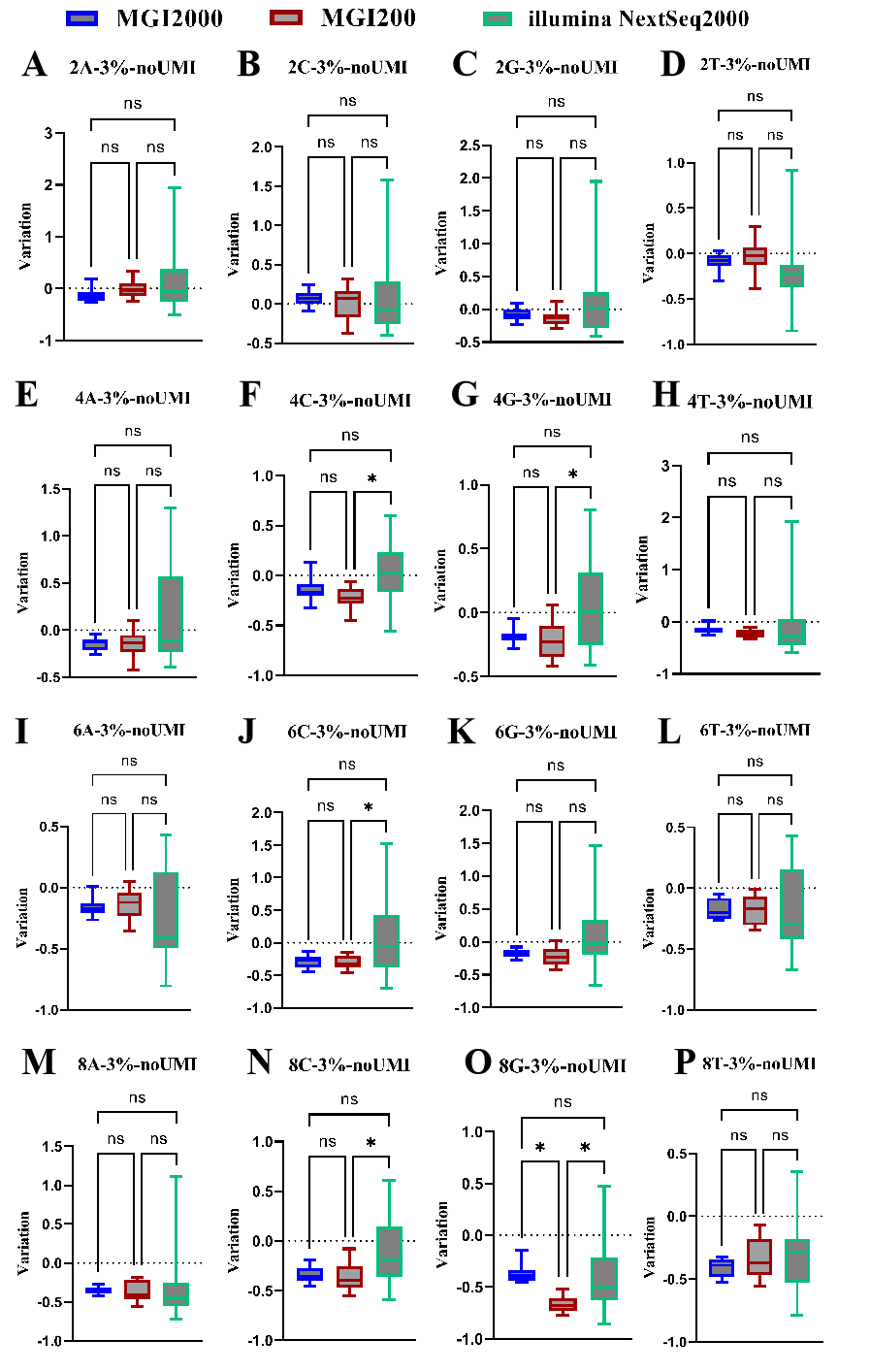

Supplement: Supplementary file 4 — Supplementary Material 4 [file 12864_2024_10474_MOESM4_ESM.tif]

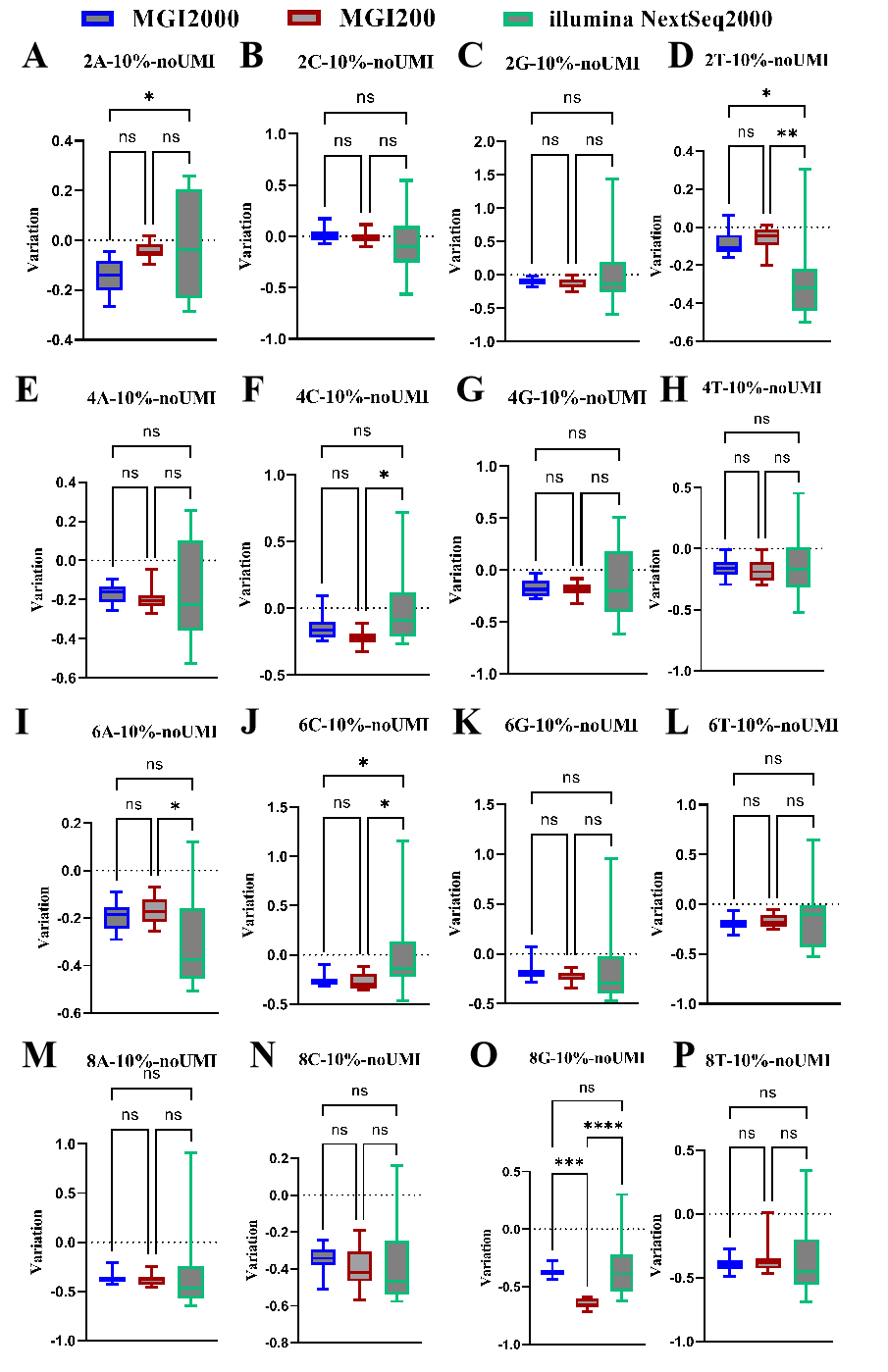

Supplement: Supplementary file 5 — Supplementary Material 5 [file 12864_2024_10474_MOESM5_ESM.tif]

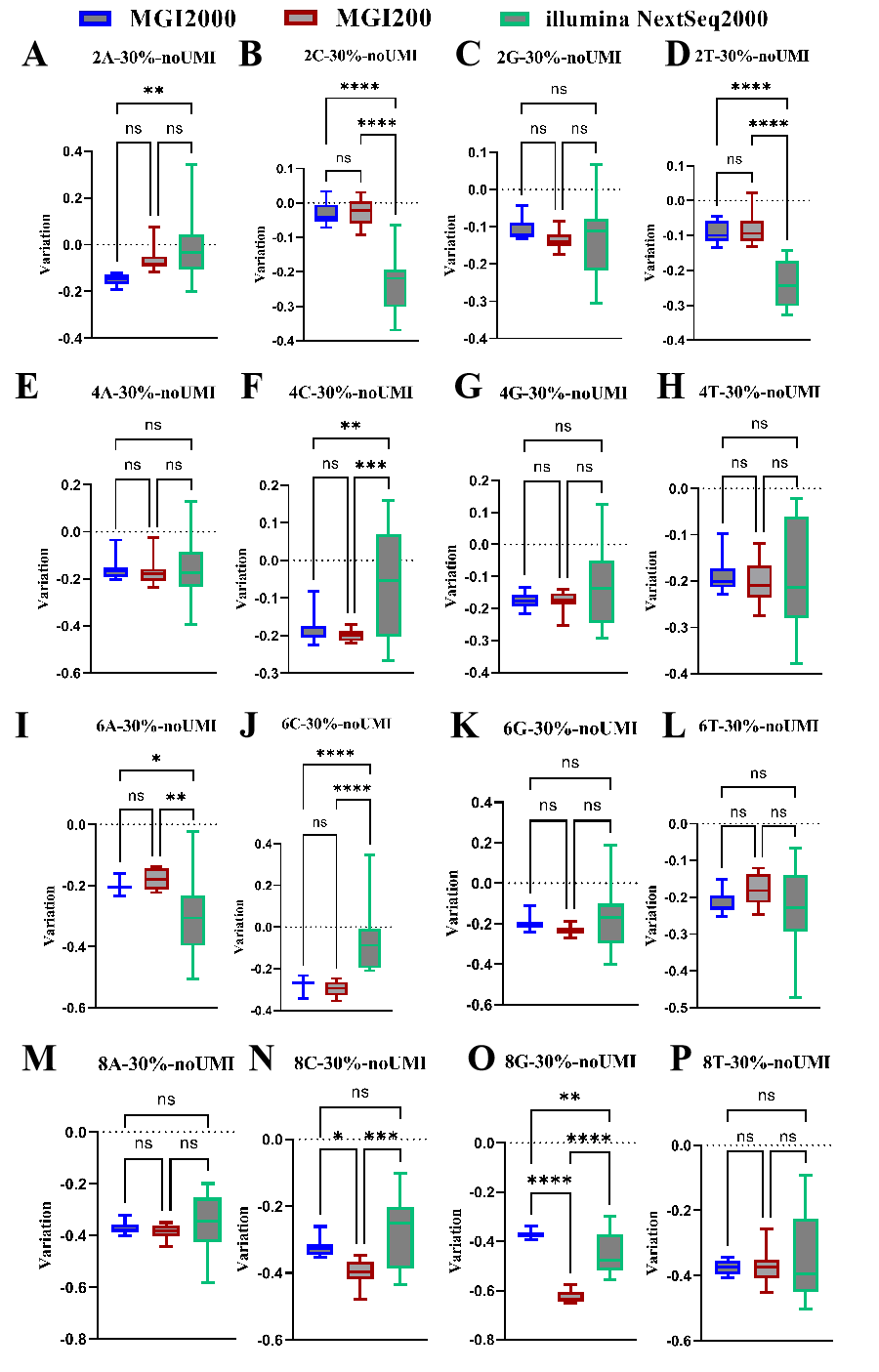

Supplement: Supplementary file 6 — Supplementary Material 6 [file 12864_2024_10474_MOESM6_ESM.tif]

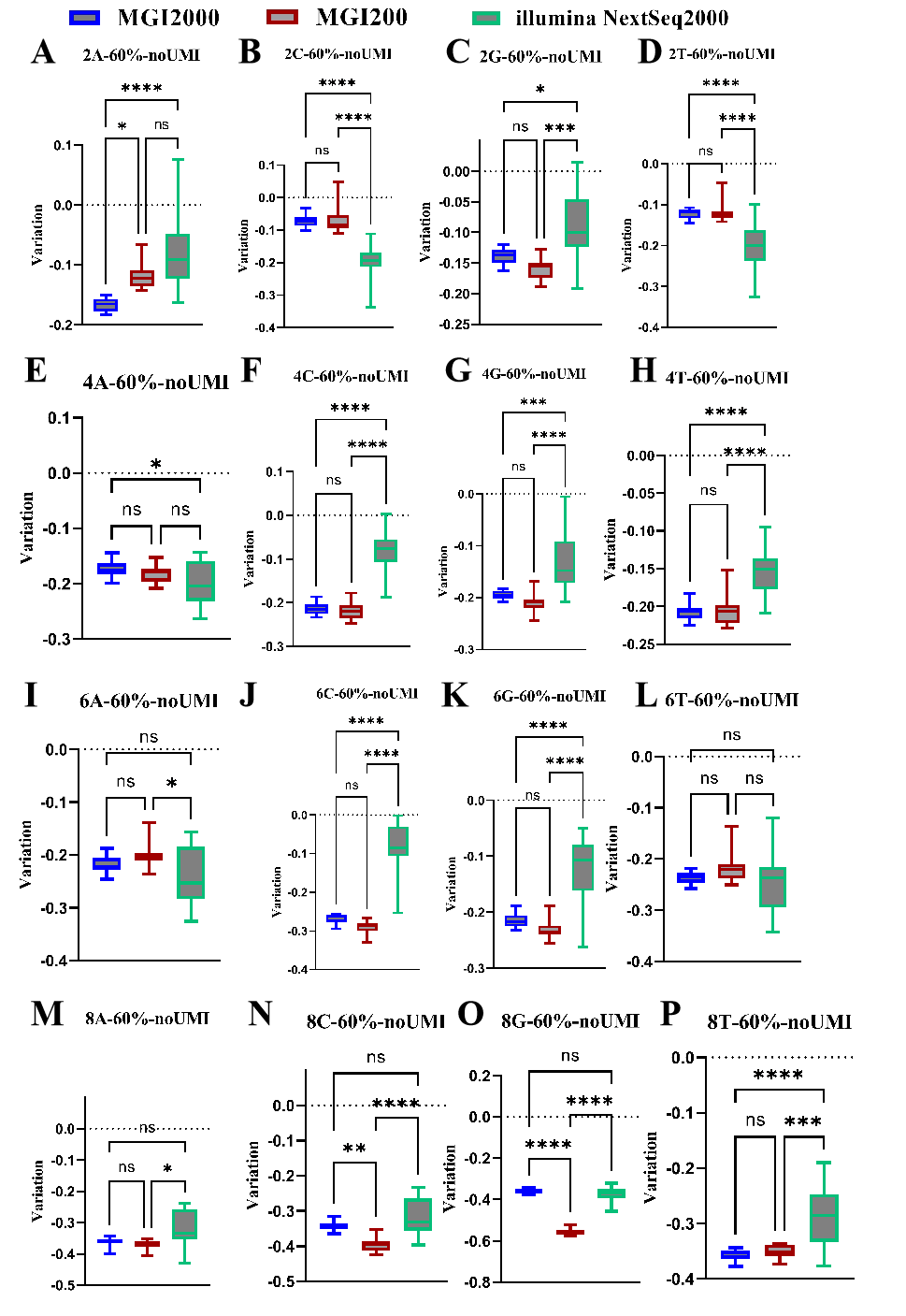

Supplement: Supplementary file 7 — Supplementary Material 7 [file 12864_2024_10474_MOESM7_ESM.tif]

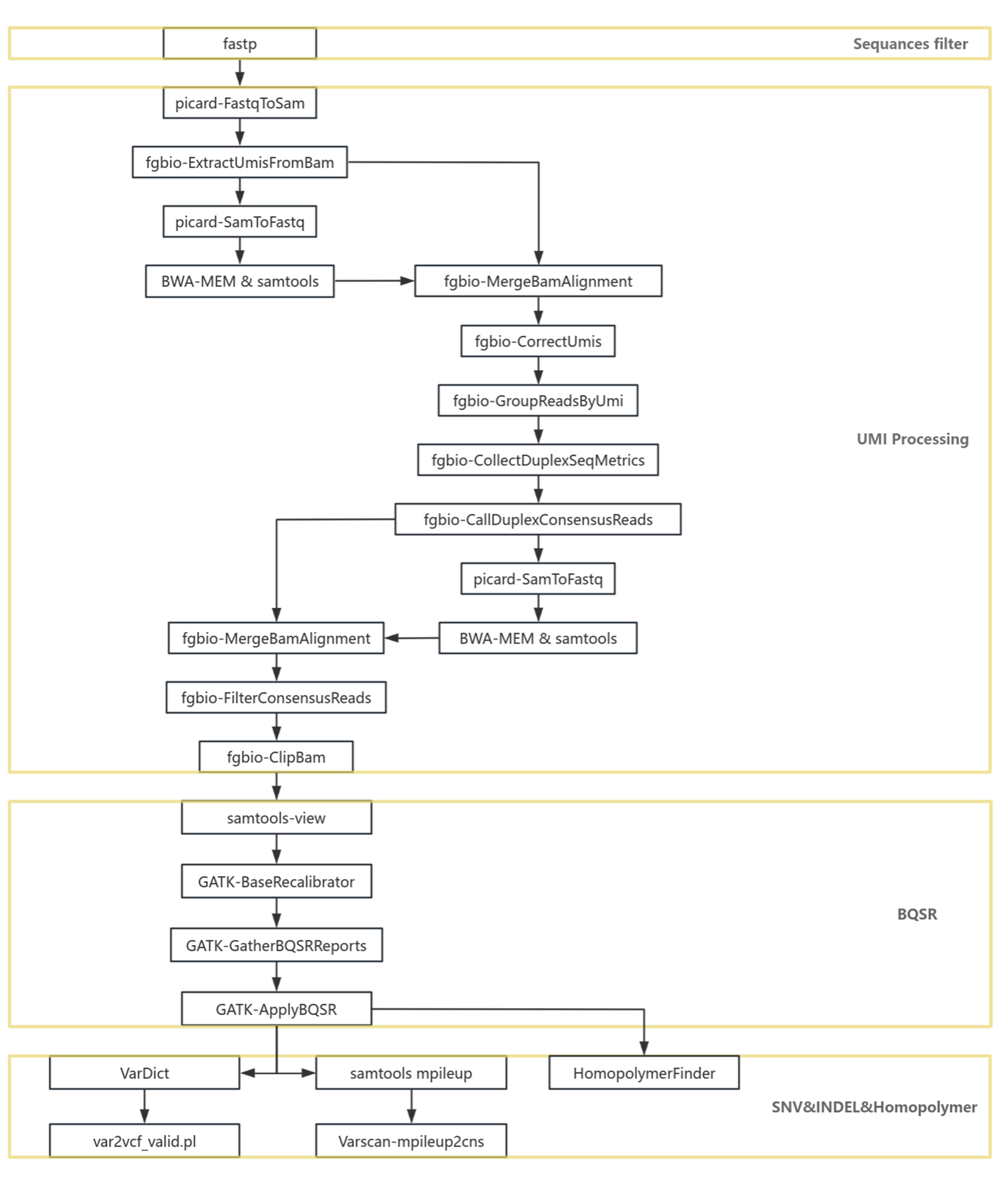

Supplement: Supplementary file 9 — Supplementary Material 9 [file 12864_2024_10474_MOESM9_ESM.jpg]

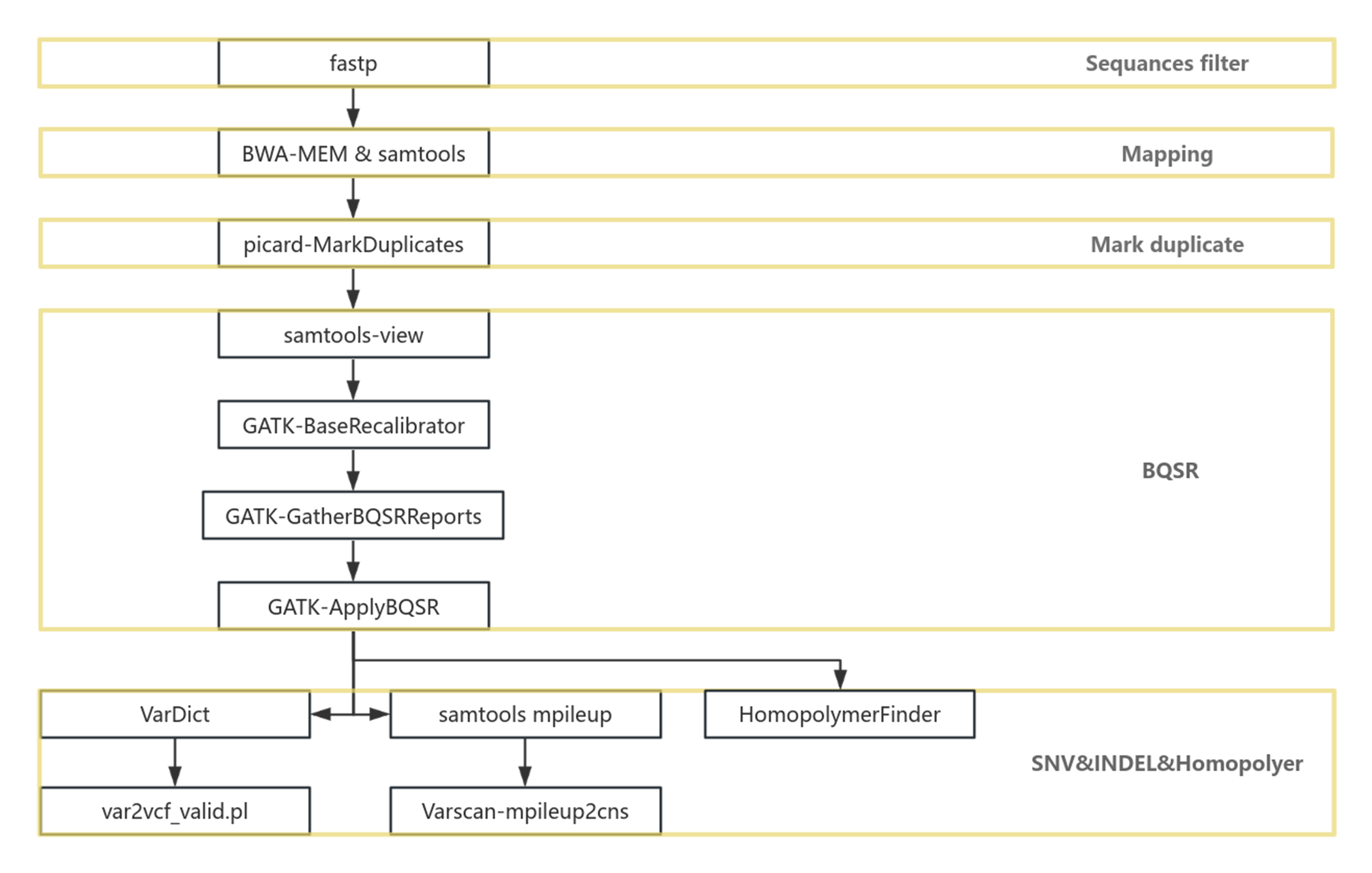

Supplement: Supplementary file 10 — Supplementary Material 10 [file 12864_2024_10474_MOESM10_ESM.jpg]
