# Supplementary material for: The performance of homopolymer detection using dichromatic and tetrachromatic fluorogenic next-generation sequencing platforms
Source: BMC Genomics. 2024 May 31;25:542. doi: 10.1186/s12864-024-10474-0 (PMC11140927; doi:10.1186/s12864-024-10474-0)
Supplement: Supplementary file 8 — Supplementary Material 8 [file 12864_2024_10474_MOESM8_ESM.docx]

**Supplementary materials:**


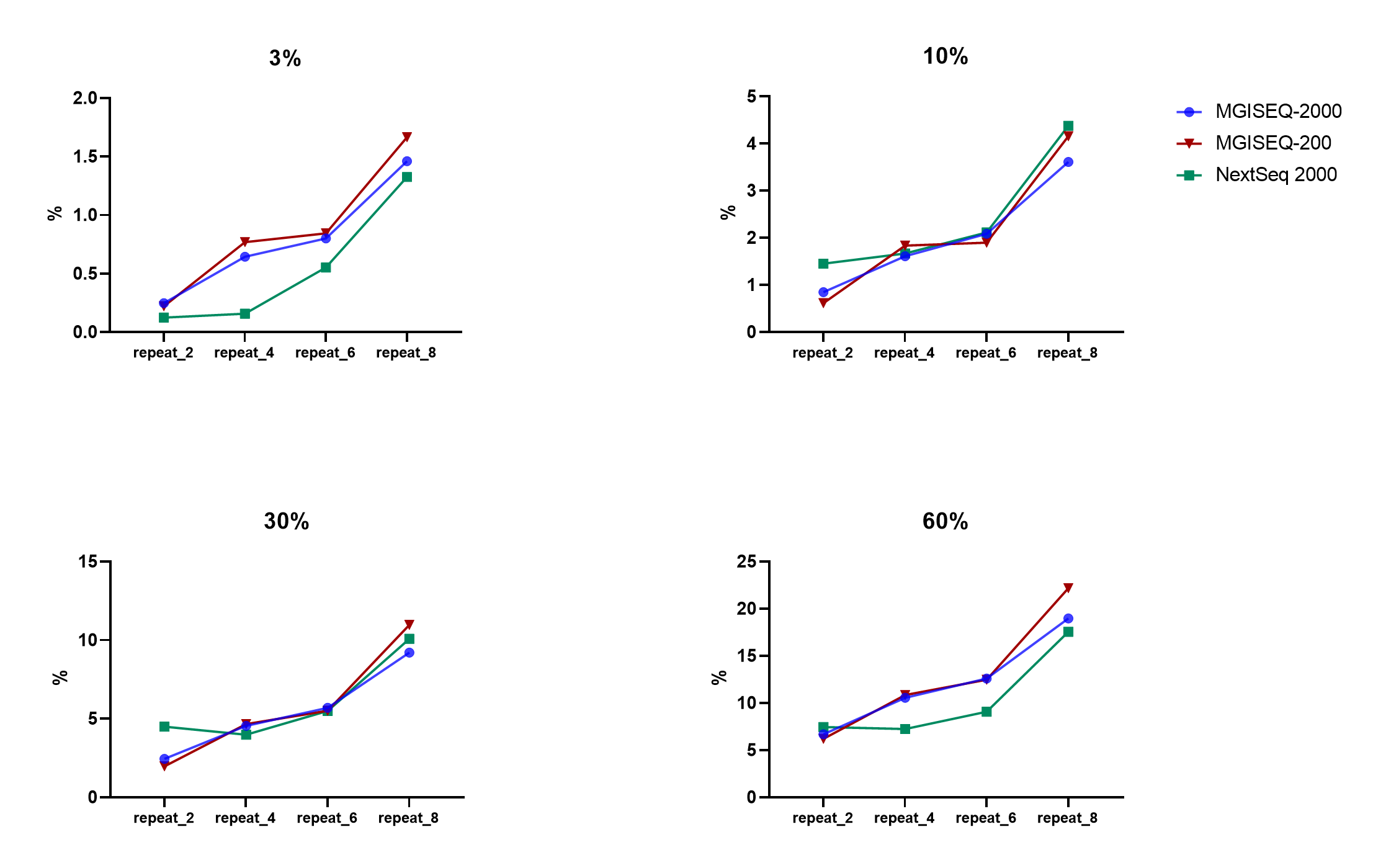


**Figure S1 Rate of homopolymeric regions which had incorrect calling**

%=Reads of incorrect calling/ total reads of homopolymeric regions


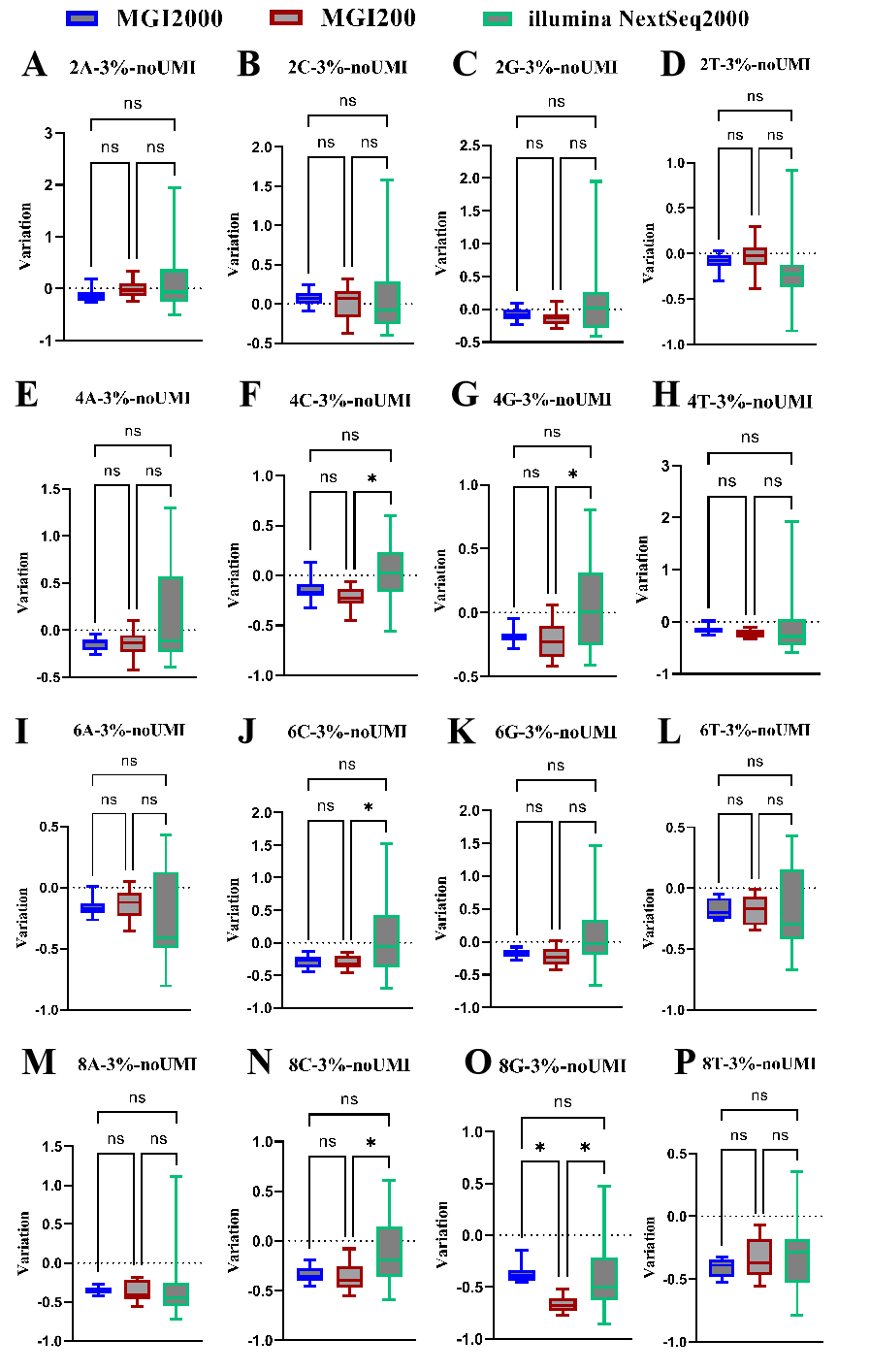


Figure S2 Performance comparison of three NGS platforms for homopolymer sequencing at 3% theoretical frequency


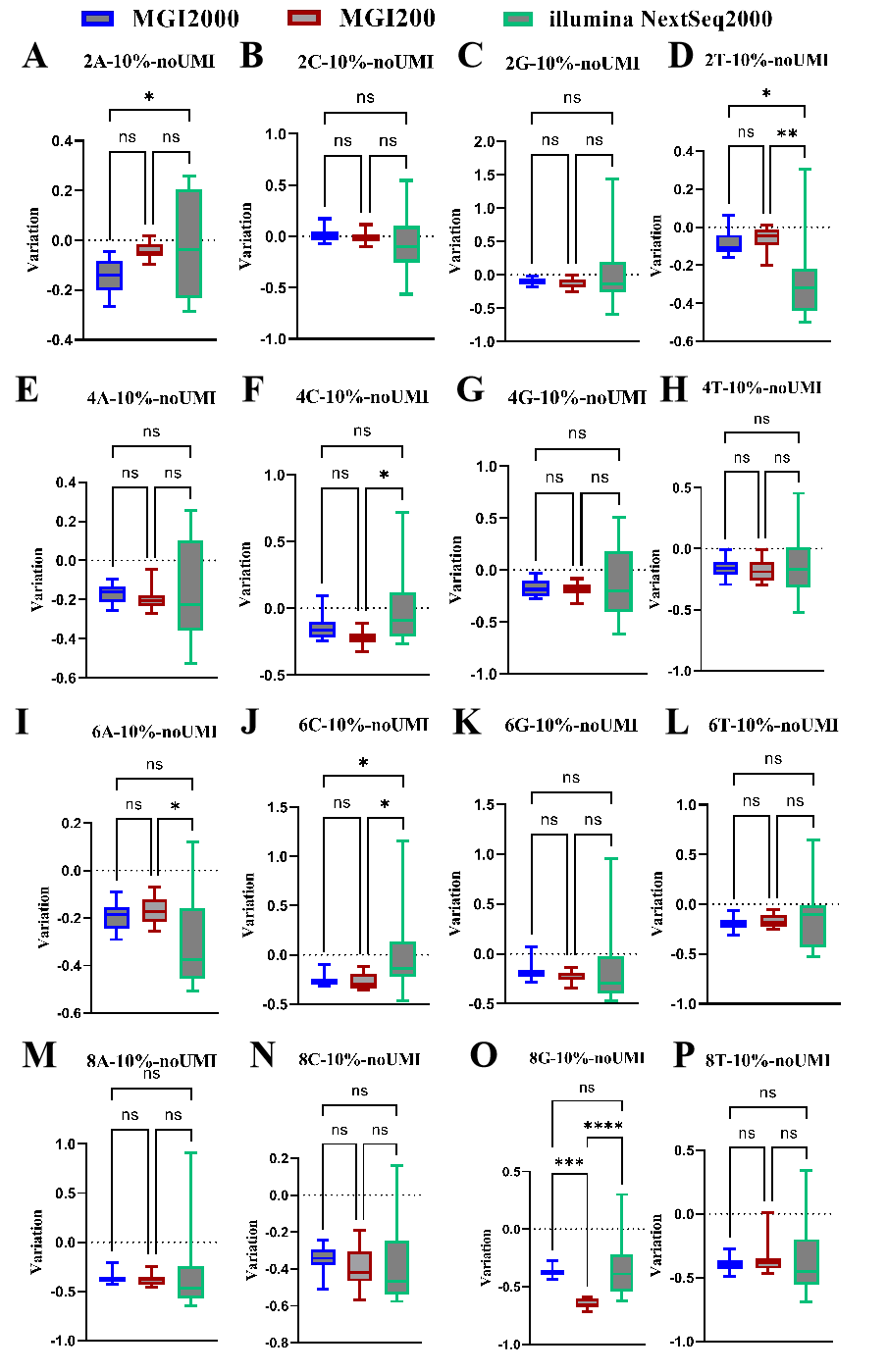


Figure S3 Performance comparison of three NGS platforms for homopolymer sequencing at 10% theoretical frequency


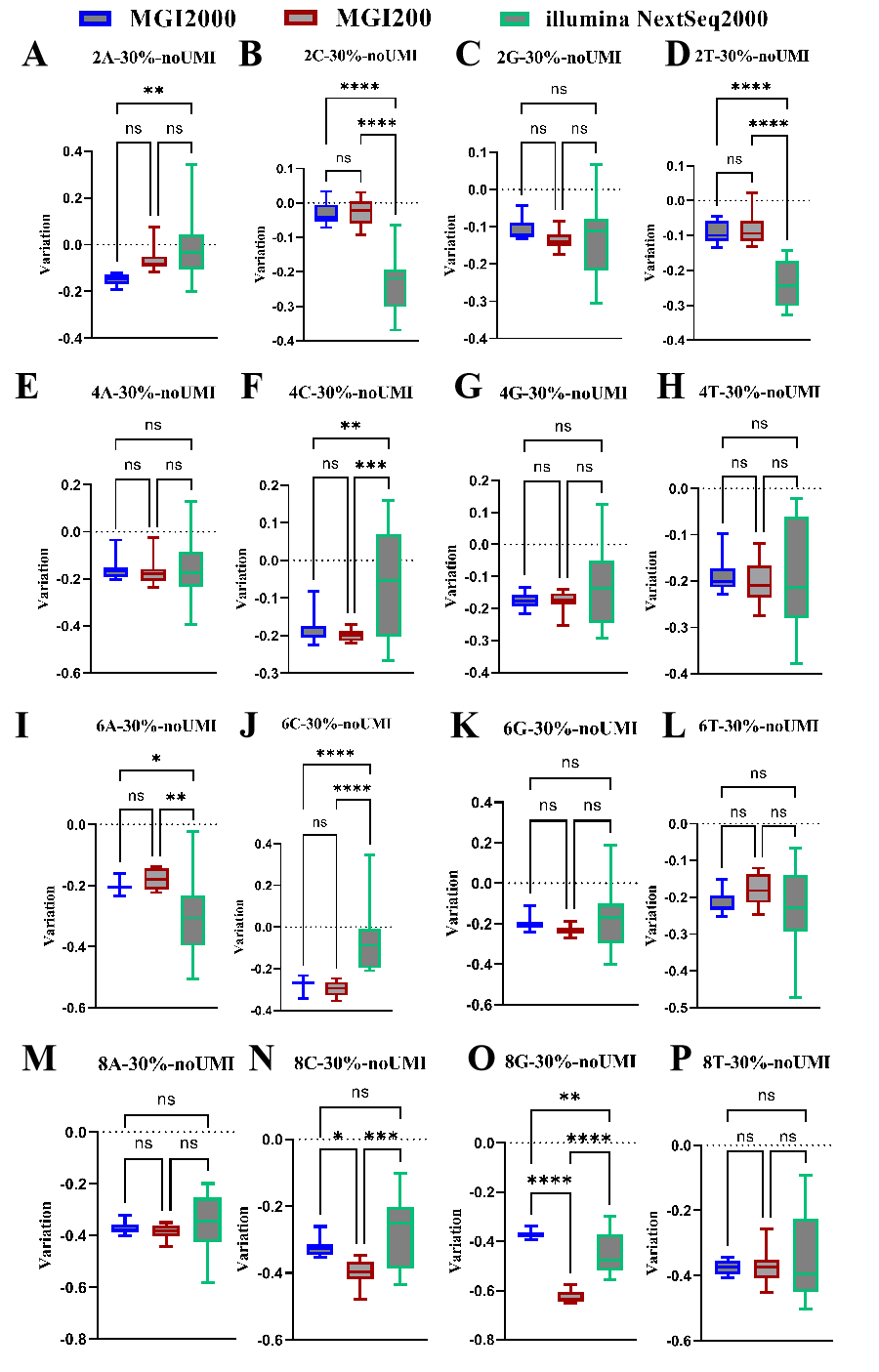


Figure S4 Performance comparison of three NGS platforms for homopolymer sequencing at 30% theoretical frequency


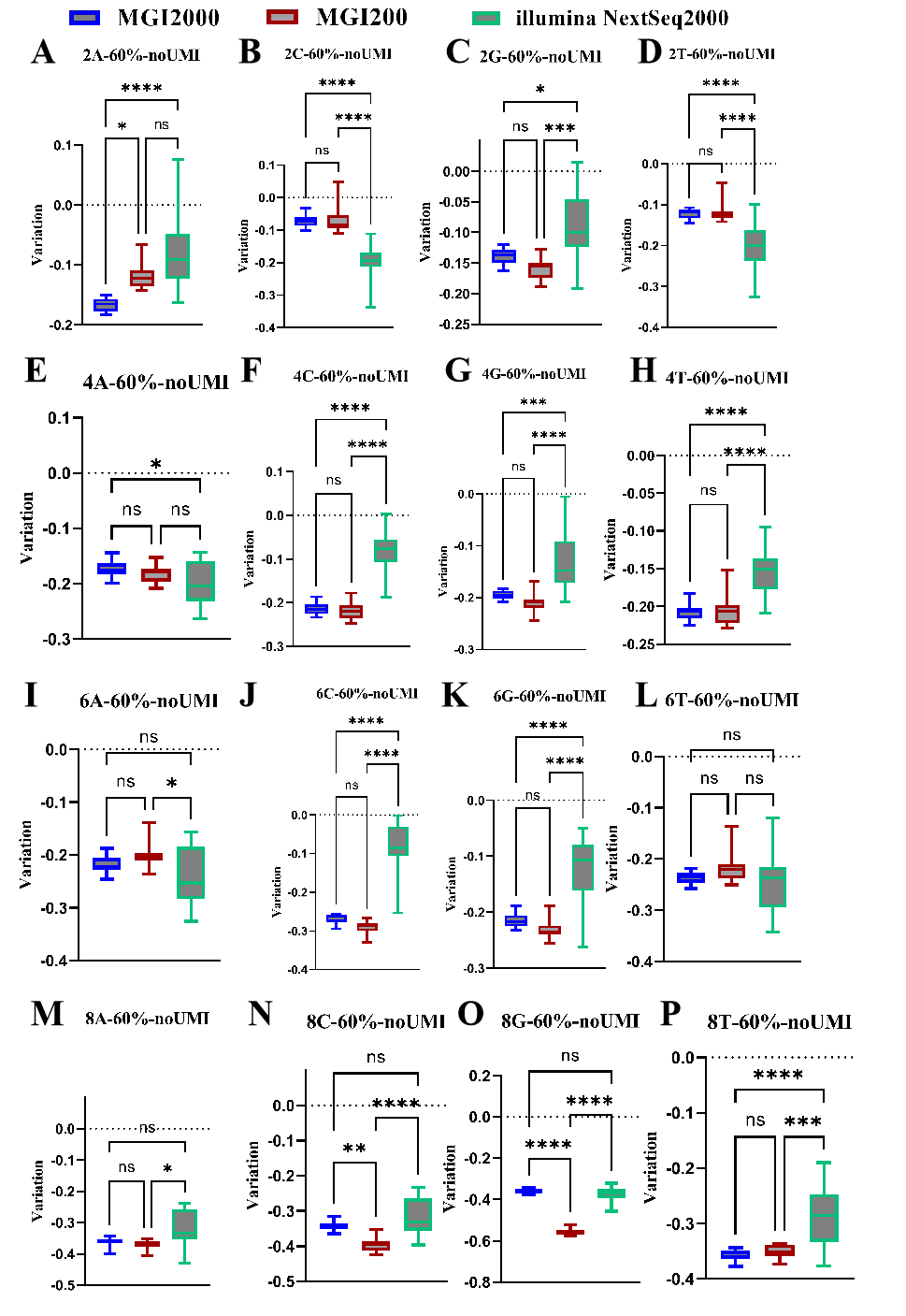
Figure S5 Performance comparison of three NGS platforms for homopolymer sequencing at 60% theoretical frequency


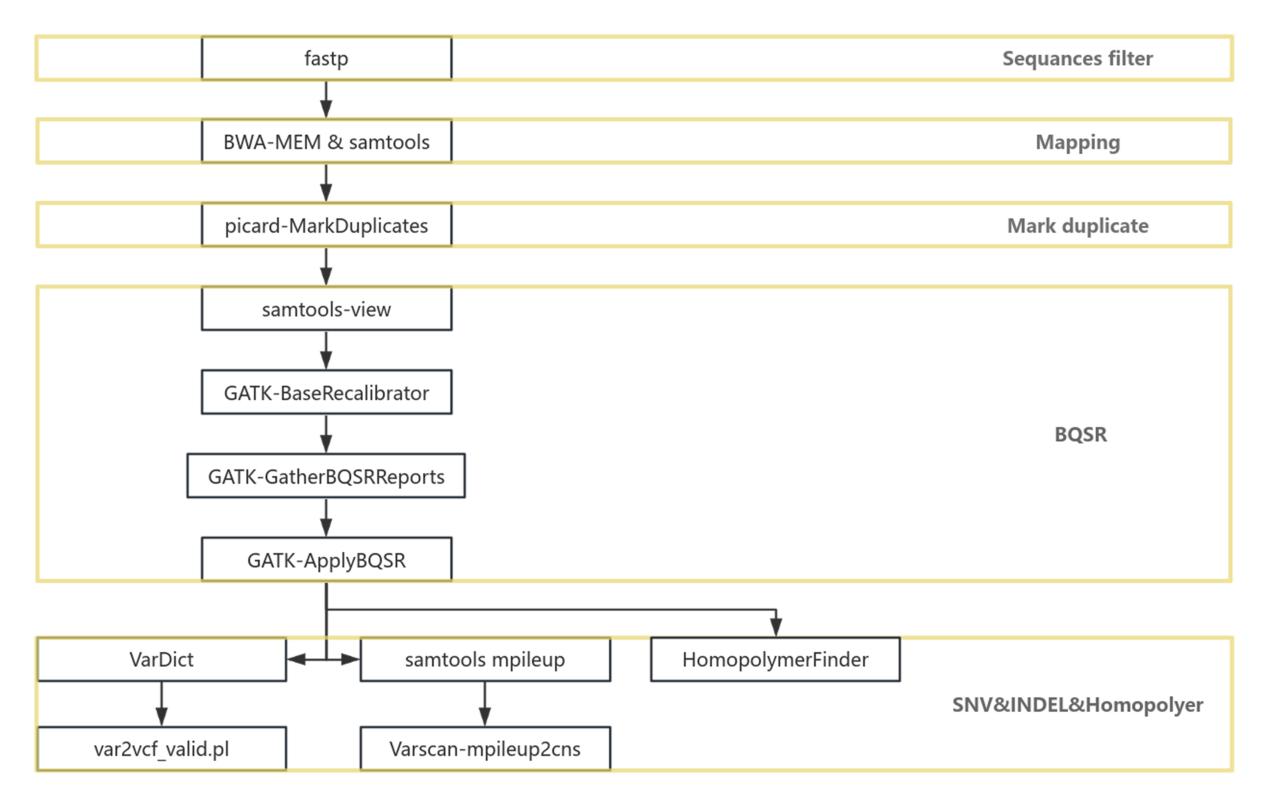


**Figure S6 The** **flow diagram of bioinformatic pipeline without UMI**


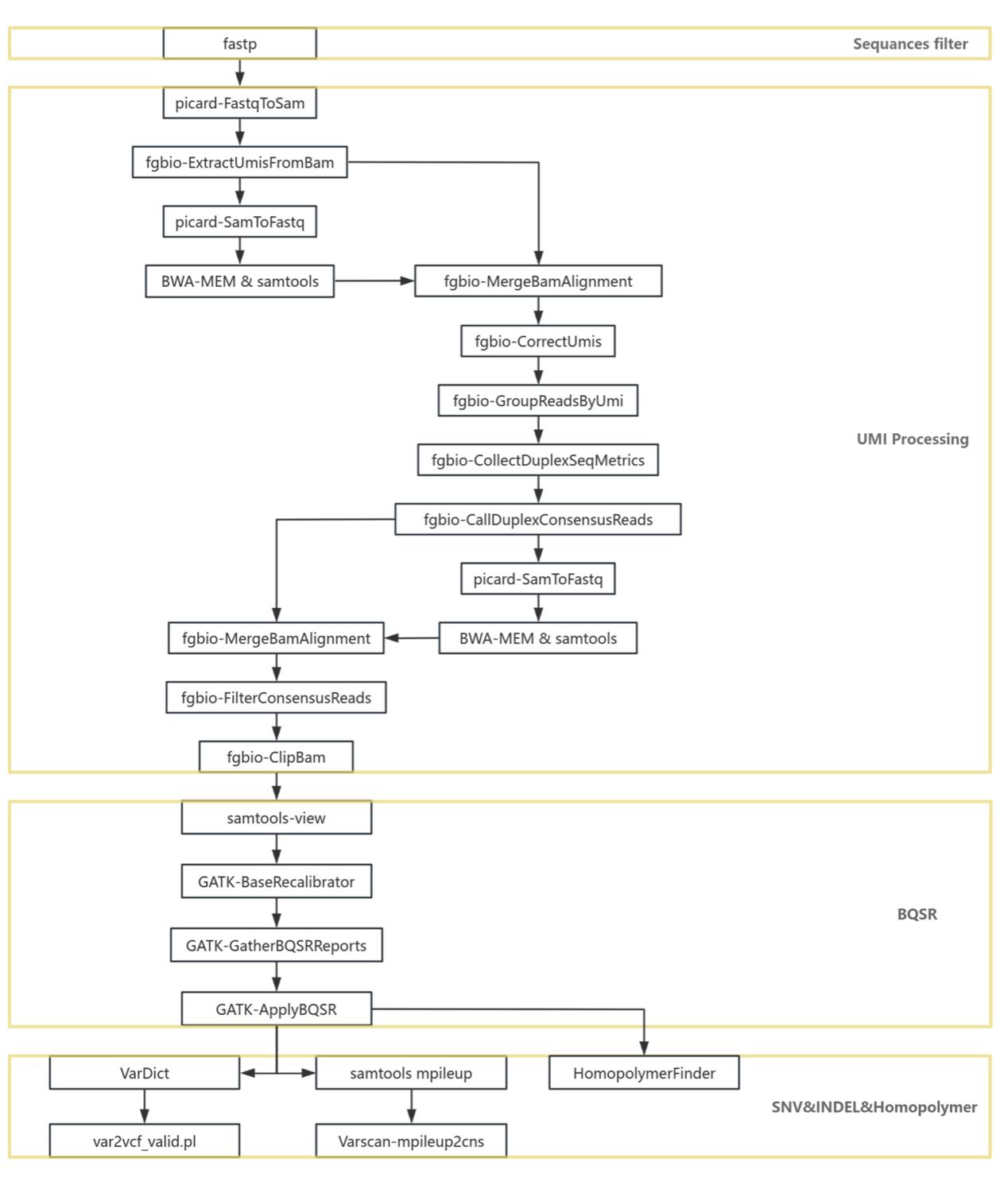


**Figure S7 The flow diagram of bioinformatic pipeline with UMI**
